# Using stochastic language models (SLM) to map lexical, syntactic, and phonological information processing in the brain

**DOI:** 10.1371/journal.pone.0177794

**Published:** 2017-05-18

**Authors:** Alessandro Lopopolo, Stefan L. Frank, Antal van den Bosch, Roel M. Willems

**Affiliations:** 1 Centre for Language Studies, Radboud University Nijmegen, Nijmegen, the Netherlands; 2 Donders Institute, Radboud University Nijmegen, Nijmegen, the Netherlands; 3 Meertens Institute, Royal Netherlands Academy of Science and Arts, Amsterdam, the Netherlands; 4 Max Planck Institute for Psycholinguistics, Nijmegen, the Netherlands; University of Akron, UNITED STATES

## Abstract

Language comprehension involves the simultaneous processing of information at the phonological, syntactic, and lexical level. We track these three distinct streams of information in the brain by using stochastic measures derived from computational language models to detect neural correlates of phoneme, part-of-speech, and word processing in an fMRI experiment. Probabilistic language models have proven to be useful tools for studying how language is processed as a sequence of symbols unfolding in time. Conditional probabilities between sequences of words are at the basis of probabilistic measures such as surprisal and perplexity which have been successfully used as predictors of several behavioural and neural correlates of sentence processing. Here we computed perplexity from sequences of words and their parts of speech, and their phonemic transcriptions. Brain activity time-locked to each word is regressed on the three model-derived measures. We observe that the brain keeps track of the statistical structure of lexical, syntactic and phonological information in distinct areas.

## 1 Introduction

“*Ze staat stil en kijkt een poosje naar een punt in de verte*” (She stands still and looks for a moment at a point in the distance) is a fluent grammatical sentence in Dutch. It consists of a string of symbols that when spoken, unfolds in time. When tokenized, it is composed of 13 word forms, belonging to seven distinct grammatical categories, and 40 phonemes (see Section 2.4). Although a natural language sentence is a linear sequence of surface forms, it allows to be decomposed into different levels of information at the phrasal, word, and sub-word level. These levels of information are what we might call, following traditional linguistic schools: syntax, lexico-semantics, and phonology [1, 2]. The sentence as a sequence of words co-exists with the sentence as a sequence of phonemes and the sentence as a sequence of grammatical categories. Models of language processing typically decompose language into these co-existing levels of information [3]. These different levels are probably processed at least partially separately by the language faculty [4, 5]. It is therefore no surprise that the study of the neural basis of language comprehension has adhered to studying one of these types of information at a time. That is, there are dedicated studies investigating syntactic aspects of comprehension, lexico-semantic aspects of comprehension, phonological aspects of comprehension, and so on (the recent textbook by Kemmerer offers a clear overviews of the state of the art in each subdiscipline in the cognitive neuroscience of language [6]). Here, we aim to investigate the neural basis of the three streams of information processing during language comprehension simultaneously, within one experiment. In other words, we want to know whether we can fractionate the neural signature of language processing of natural speech in different, parallel streams within one and the same data set.

In order to do so, we fitted characterizations of lexical, phonological and syntactic information to fMRI data collected while participants listened to narratives. Brain activity time-locked to each word is regressed on the three model-derived measures. Stochastic language models computed at the word, part-of-speech and phoneme level are used to operationalize the concept of parallel lexical, syntactic and phonetic streams. We rely on stochastic models because, as detailed in Section 1.1, they have been proven powerful tools for the study of language comprehension. Such an approach goes beyond the typical studies in which one level of processing is studied per experiment.

We begin with a short overview of work in modelling language comprehension using stochastic language models in Section 1.1. In Section 1.2 we introduce the *Streams of processing* framework. In section 2 we present the methodological details of the study, focusing both on the stochastic language models and the computational linguistic techniques employed to annotate and extract lexical, syntactic and phonological information from a common stimulus. Section 2 also provides details on the fMRI data collection paradigm. Section 3 describes the regression analysis employed to delineate the neural correlates of each level of information. Finally, in Sections 4 and 5 we report the results and draw conclusions according to the hypotheses expressed in the previous sections.

### 1.1 Language processing as a sequential stochastic process

A number of studies [7–11] have advanced the hypothesis that the brain employs predictive coding strategies in perception. The hypothesis is that after processing the first *t* − 1 elements of a sequence of stimuli (i.e., *x*_1_, …, *x*_*t*−1_), the human brain assigns a conditional probability *P*(*x*_*t*_|*x*_1_, …, *x*_*t*−1_) to each potential element *x* that can follow at time *t*. These expectations influence the way the actual observed *x*_*t*_ is processed eventually. Deviations from expectations are usually quantified in terms of surprisal or perplexity, which have been shown to explain both behavioural and neural correlates of perceptual and higher cognitive processing.

In the domain of language processing, word surprisal has been used to predict a wide range of behavioural correlates. It has been found to predict the duration of spoken words, with shorter words being used in less surprising situations [12, 13]. Following Hale [14] and Levy [15], surprisal has been hypothesized to be proportional to the cognitive effort required to integrate a word into the current context. This has been confirmed by observing that it correlates with reading times [16–19]. Reading time has also been shown to be correlated to surprisal of the syntactic category (part-of-speech; PoS) of the word being read [20, 21]. Moreover, Monsalve [17] showed that PoS and word surprisal have independent effects on reading times. This behavioural result suggests that the PoS of words in sentential context is a valid representation of linguistic information relevant for processing, and that computing probabilistic measures on them returns a model that has significant predictive power. These measures have also been successfully applied to the prediction of brain activity. It was found that the amplitude of the N400 event-related potential (ERP) component elicited by words in sentences correlates with word surprisal values [22, 23]. The fact that surprisal correlates with the amplitude of a classical ERP component related to language comprehension [24] is another source of evidence that stochastic language modelling is a neuro-cognitively valid approximation of sentence comprehension. In a recent paper, Willems and colleagues [25] applied surprisal and entropy to an fMRI dataset to predict brain activity in different cortical and subcortical areas during naturalistic language comprehension. They observed that different areas differentially code for statistical stimulus properties by selectively correlating with one or the other measure.

### 1.2 Probabilistic streams

Our starting point is twofold. On one hand, we hypothesize that different types of information correspond to different streams of processing implemented in separable networks in the brain. Several linguistic models, for instance, separate phonological, semantic and syntactic processing in different neural loci or processing streams [6]. One way to operationalize this search for parallel streams is to model the processed linguistic input as composed by three parallel levels of representation corresponding to its phonological, lexical and syntactic profile. On the other hand, following the findings exposed in section 1.1, we model the processing in these separate streams as sequential and incremental, and sensitive to the stochastic properties of the information it is applied to. The difference between different streams is that the probabilistic relations are computed not only on surface forms (bare words, so to speak) but on the phonemic transcription and the grammatical categories of the words in order to disentangle the different levels of representation.

The fact that language can be studied as a stochastic process does not necessarily mean that subcomponents of language correspond to distinct stochastic processes that are detectable in the brain. One scenario could be that the areas sensitive to stochastic properties of the input are the same, independently from the level of annotation on which such measures have been computed. In a recent study, Nastase and colleagues [26] investigated whether there exist areas in the brain that are sensitive to probabilistic properties of the incoming signal, independently from its sensory modality, or if, conversely, sensitivity to such properties is an intrinsic characteristic of domain-specific areas. Their approach consisted in looking for areas coding for the degree of disorder—quantified by Markov entropy—in a temporally unfolding sensory input of two distinct modalities: auditory and visual. Their results show a modality specific sensitivity to input entropy, implemented in modality-specific systems of sensory cortices (for visual stimuli: the early visual cortex, the anterior cingulate, and the intraparietal sulcus; for acoustic stimuli: inferior frontal, lateral temporal, and supplementary motor regions). Ventral premotor and central cingulate cortices were identified as possible candidates for modality-general uncertainty processing, exhibiting sensitivity to disorder in both modalities.

We approach the problem of disentangling phonology, lexical and syntax by using language stimuli which are not explicitly designed to study one of these levels in isolation [22, 25]. Using one stochastic measure computed on three distinct levels of annotations of the same linguistic stimulus, we want to investigate first of all level-specific processing, what we may refer to as streams of information. On the other hand, we are interested in investigating the issue of whether there exists a central supramodal stochastic processor of the brain (what is called modality-independent in [26]) by finding areas that are sensitive to stochastic measures independently from the level of information they have been computed on.

## 2 Materials and methods

Ethical approval was obtained from the CMO Committee on Research Involving Human Subjects, Arnhem-Nijmegen, The Netherlands (protocol number 2001/095), in line with the Declaration of Helsinki.

### 2.1 Participants and stimuli

We re-analysed data from an fMRI study on language comprehension of auditory presented narrative texts [25]. Here we briefly present the data collection procedure, preprocessing, and stimuli employed. Full details can be found in the original paper.

Twenty-four healthy, native speakers of Dutch (8 males; mean age 22.9, range 18–31) without psychiatric or neurological problems, with normal or corrected-to-normal vision, and without hearing problems took part in the experiment. All participants except one were right-handed by self-report, and all participants were naive with respect to the purpose of the experiment. Written informed consent was obtained in accordance with the Declaration of Helsinki, and the study was approved by the local ethics committee. Participants were paid either in money or in course credit at the end of the study.

Stimuli consisted of three excerpts from three distinct literary novels extracted from the Spoken Dutch Corpus, “Corpus Gesproken Nederlands” (CGN) [27]. The excerpts were spoken at a normal rate, in a quiet room, by female speakers (one speaker per story). Stimulus durations were 3:49 min (622 words), 7:50 min (1,291 words), and 7:48 min (1,131 words). Reversed speech versions of the stories were created with Audacity 2.03 (http://www.audacityteam.org/).

### 2.2 Procedure

The experimental paradigm consisted in passively listening to the three narratives and their reversed versions (for a total of six sessions) inside the MRI scanner. Each story and its reversed speech counterpart were presented following each other. Half the participants started with a non-reversed stimulus, and half with a reversed speech stimulus. Participants were instructed to listen to the materials attentively, which in practice is only possible for three narratives, and not for the reversed speech counterparts. There was a short break after each fragment.

Stimuli were presented with Presentation 16.2 (https://www.neurobs.com/). Auditory stimuli were presented through MR-compatible earphones. In order to make sure participants could correctly perceive the stimuli, the actual experimental sessions were preceded by an in-scanner volume test where a fragment from another story with comparable voice and sound quality was presented and the volume was adjusted to the optimal level based on feedback from the participant.

After the scanning session, participants were tested for their memory and comprehension of the stories. The participants were not informed in advance about the test in order to avoid attentional biases during the passive listening to the stories.

### 2.3 fMRI data acquisition and preprocessing

Images of blood-oxygenation level-dependent (BOLD) changes were acquired on a 3-T Siemens Magnetom Trio scanner (Erlangen, Germany) with a 32-channel head coil. Pillows and tape were used to minimize participants’ head movement, and the earphones that were used for presenting the stories reduced scanner noise. Functional images were acquired using a fast T2*-weighted 3D echo planar imaging sequence [28], with high temporal resolution (time to repetition: 880 ms, time to echo: 28 ms, flip angle: 14°, voxel size: 3.5 × 3.5 × 3.5 mm, 36 slices). High resolution (1 × 1 × 1.25 mm) structural (anatomical) images were acquired using a T1 sequence.

Preprocessing was performed using SPM8 (http://www.fil.ion.ucl.ac.uk/spm) and Matlab 2010b (http://www.mathworks.nl/). The first four volumes were removed to control for T1 equilibration effects. Rigid body registration was used to realign images. Images were realigned to the first image within each run. The mean of the motion-corrected images was then brought into the same space as the individual participant’s anatomical scan. The anatomical and functional scans were spatially normalized to the standard MNI template, and functional images were re-sampled to 2 × 2 × 2 mm voxel sizes. Finally, an isotropic 8-mm full-width at half-maximum Gaussian kernel was used to spatially smooth the motion-corrected and normalized data.

### 2.4 Estimation of stream-wise stochastic properties

The three levels of information –phonological, syntactic and lexical– are distinguished by applying three different levels of annotation to the stimulus narratives. At the phonological level, the words in the running text are transformed into a sequence of phonemes. The syntactic level is approximated by the sequence of fine-grained syntactic categories corresponding to the words of the texts, also known as parts of speech (PoS). The lexical level consists of the sequence of surface lexical forms composing the texts. In the sections below we describe the characteristics of each stream and their common computational properties.

The lexical level is the sequence of words constituting the sentences of the book fragments. At this level, a sentence can be rewritten as a sequence *w*_1_, …, *w*_*n*_ of symbols *w*_*i*_ belonging to the vocabulary V containing all the word form, as illustrated in Table 1.

**Table 1:** The lexical stream is obtained from the simple sequence of word forms in the stimulus presented to the subjects.

Ze	staat	stil	en	kijkt	een	poosje	naar	een	punt	in	de	verte
*w*_1_	*w*_2_	*w*_3_	*w*_4_	*w*_5_	*w*_6_	*w*_7_	*w*_8_	*w*_9_	*w*_10_	*w*_11_	*w*_12_	*w*_13_

The phonological stream can be defined as the sequence of phonemes composing each single word in the sentence. Therefore, the sentence in Table 1 can be rewritten as a sequence $p_{1}^{1},p_{1}^{2},...,p_{1}^{m},...,p_{13}^{1},...,p_{13}^{o}$ where $p_{j}^{i}$ refers to the *i*^th^ phoneme of the *j*^th^ word in the sentence. Table 2 contains the phonetic transcription of the example sentence already presented in Table 1.

**Table 2:** The phonological stream is obtained from the phonetic transcription of the words of the stimulus.

Ze	staat	stil	en	kijkt	een	poosje	naar	een	punt	in	de	verte
[zə]	[stat]	[stɪl]	[ɛn]	[kɛɪkt]	[ən]	[poʃə]	[nar]	[ən]	[pynt]	[ɪn]	[də]	[vɛrtə]

Finally, the words in the stimuli are assigned with their syntactic categories, or part of speech tags (PoS). Parts of speech are a basic ingredient of most language technology systems and act as shallow (i.e., non-hierarchical) syntactic starting point for many other tasks, including semantic role assignment and dependency and constituent syntactic parsing. They usually consist of a basic set of grammatical categories such as nouns (N), verbs (WW, in the Dutch tags used here), modifiers and determiners. They capture, when considered in context, shallow, yet robust, combinatorial constraints that abstract away from the lexical information within the surface forms. The tagset employed here was the one employed by CGN (the corpus from which the stimuli for our experiments were taken) and comprises 320 tags (see Table 3). Besides 13 base tags, this method explicitly assign morpho-syntactic sub-category features to the base tags containing information such as gender, number, form and so on.

**Table 3:** Summary of the types of grammatical categories (POS) and the number of sub-categories used to approximate sequential syntactic information processing.

Dutch POS	tag	#	English equivalent	Example
substantieven	N	18	Nouns	het kind
adjectieven	ADJ	30	Adjectives	de mooie huizen
werkwoorden	WW	21	Verbs	ik kom
telwoorden	TW	11	Quantifiers	vier cijfers
voornaamwoorden	VNW	188	Pronouns	ik
lidwoorden	LID	9	Articles	de hond
voorzetsels	VZ	3	Prepositions	in het hospitaal
voegwoorden	VG	2	Conjunctions	Jan en Peter
bijwoorden	BW	1	Adverbs	gisteren
tussenwerpsels	TSW	1	Interjections	hoera!
speciale tokens	SPEC	35	special forms	
leestekens	LET	1	Punctuation	.
TOTAL		320		

This tagset closely follows the practices of the Dutch Grammar ‘Algemene Nederlandse Spraakkunst’ (ANS) [29]. Table 4 contains an example of PoS annotation of the example sentence presented in Table 1 above.

**Table 4:** The same sentence from Tables 1 and 2 annotated with fine-grained grammatical information using the POS tags described above.

Ze	staat	stil	en	kijkt	een	poosje	naar	een	punt	in	de	verte
VNW	WW	ADJ	VG	WW	LID	N	VZ	LID	N	VZ	LID	N

### 2.5 Computing stochastic measures

The conditional probabilities required for obtaining perplexity values for the lexical and PoS streams are estimated by a second-order Markov model, also known as trigram model, trained on a large collection of text. It is based on the simplifying assumption that the probability of word *w*_*t*_ depends on the previous two words only, that is, *P*(*w*_*t*_|*w*_1_, …, *w*_*t*−1_) is reduced to *P*(*w*_*t*_|*w*_1_, …, *w*_*t*−1_). Surprisal is computed as the negative logarithm of the conditional probability of wt given *w*_*t*−2_, *w*_*t*−1_:

$$\text{surprisal}\left(w_{t}\right)=-\log P\left(w_{t}|w_{t-2},w_{t-1}\right)$$

If the observed word’s probability equals 1, observing it yields a surprisal of 0. Conversely, the occurrence of a word that was not among the words considered possible (i.e., has zero probability) corresponds to infinite surprisal. Surprisal can be thought of as the degree to which the actually perceived word *w*_*t*_ deviates from expectation. Perplexity, as adopted here, consists in an exponential transformation of the surprisal of encountering *w*_*t*_ given *w*_*t*−2_, *w*_*t*−1_.

$$\text{ppl}\left(w_{t}\right)=2^{\text{surprisal}(w_{t})}=2^{-\log P(w_{t}|w_{t-1})}$$

The dataset from which probabilities *P*(*w*_*t*_|*w*_1_, …, *w*_*t*−1_) are estimated is a random selection of 10 million sentences (comprising 197 million word tokens; 2.1 million types) from the Dutch Corpus of Web (NLCOW2012) [30]. For lexical perplexity, each word of the experimental texts is assigned a value computed by SRILM [31].

The PoS perplexity is computed analogously. Instead of using the surface forms of the training and stimulus set, the trigram model was trained on the PoS-tagged version of the same 10 million sentences subset of NLCOW2012. The tagging was performed using the Frog toolbox for natural language processing of Dutch text [32].

The phonological information was extracted from the phonemic transcription of each word in the stimulus set. We used a memory-based grapheme phoneme converter [33] trained on CELEX 2 [34]. Once every word is transcribed as a sequence of phonemes, trigrams were extracted and conditional probabilities *P*(*p*_*t*_|*p*_*t*−1_, *p*_*t*−2_) were computed using WOPR (https://ilk.uvt.nl/wopr/) trained on CELEX 2 [34]. Once phoneme-wise perplexity is computed, the phonetic perplexity of each word of the stimulus is computed as the average value across the phonemes of that word.

## 3 Data analysis

At the single-subject level, the observed BOLD time course in each voxel is subjected to a regression analysis, testing for voxels in which the covariates of interest (word, PoS, and phonological perplexity) explain a significant proportion of variance of that voxel’s time course [35]. Before the actual analysis, one regressor modelling the duration of each single word was created for each story. This regressor was convolved with the hemodynamic response function, to account for the delay in BOLD activation respective to stimulus presentation. The word duration regressor and the covariates for a story were also fitted to the data of the reversed speech version of that story. This served as a control condition since the regressors and covariates are essentially meaningless for the reversed speech data. Three covariates were computed containing each word’s word, PoS and phonemic perplexity measures, constituting our regressors of interest modelling the three information streams introduced above. Besides these, log2-transformed lexical frequency per word was computed using the Subtlex NL corpus [36], log2-transformed PoS frequency per word was computed using the CGN corpus [27], and log2-transformed phoneme frequency average per word was computed using CELEX 2 [34]. They were used as regressors of no interest to statistically factor out effects of word, PoS and phoneme frequency. The estimates from the motion correction algorithm (three rotations and three translations per run) were additionally added as regressors of no interest.

The modelled time courses from all six runs (three stories and three reversed speech stimuli) were combined in one regression model, with separate constant terms per run, but the same regressors for real and reversed speech. The analyses were conducted at the whole-brain level. The difference in the effect of the regressor of interest between the real and reversed speech sessions was used as input to the group-level statistics. Statistical differences were assessed by computing the t-statistic over participants of this difference score (real vs. reversed speech) for each voxel in the brain. The resulting multiple comparisons problem was solved by means of combining a *P* < 0.005 voxel threshold with a cluster extent threshold determined by means of 1,000 Monte Carlo simulations, after estimation of the smoothness of the data applied for each separate contrast. The combination of a voxel level threshold with a cluster extend threshold is a good compromise between statistical sensitivity on the one hand and false positive error control on the other hand [37, 38]. The simulations took the amount of autocorrelation in the data into account, as suggested in the literature [37, 38]. The scripts used were taken from (https://www2.bc.edu/~slotnics/scripts.html). Table 5 reports the size thresholds for each regressor contrast separately. All reported clusters of size display results significant at the *P* < 0.05 level, corrected for multiple comparisons.

**Table 5:** Cluster size thresholds for the perplexity-based regressors.

Region	Cluster size
Word-based perplexity	92
POS-based perplexity	92
Phoneme-based perplexity	97

### 3.1 Relation between the regressors

The aim of this study is to assess whether different types of linguistic information can be traced in the brain, and if this can be achieved by using stochastic measures of perplexity in line with the predictive brain hypothesis. In order to assess whether word, PoS, and phoneme perplexity capture different kinds of information we conducted a preliminary analysis consisting in computing their pairwise correlations. Table 6 reports these correlations (Pearson’s *r*). Both 3-gram perplexity (ppl) and 1-gram frequency (freq) computed at lexical, PoS, and phonological level are included.

**Table 6:** Correlation between the stochastic measures used in the analyses.

	Lex_ppl	PoS_ppl	Pho_ppl	Lex_freq	PoS_freq	Pho_freq
Lex_ppl	1	0.046	0.011	−0.466	−0.092	0.080
PoS_ppl		1	−0.012	−0.015	−0.491	0.000
Pho_ppl			1	−0.016	−0.000	−0.017
Lex_freq				1	0.070	−0.060
PoS_freq					1	0.105
Pho_freq						1

The correlations between perplexity measures reported in Table 6 are fairly low, even between lexical and PoS perplexity (0.046). These results indicates that there is no strong relation between the regressors we have employed in our fMRI analyses, and that they may capture different types of information. Correlation between lexical perplexity and frequency is −0.466, and correlation between PoS perplexity and frequency is −0.491. These negative correlations between perplexity and frequency measures are predictable: the less frequent an item is, the higher is the overall perplexity of encountering it.

## 4 Results

In this section we present the results of the analyses conducted using the three perplexity measures as regressors of interest. In supplementary material S2 File we report the results of a similar analysis conducted using frequencies as regressors of interest.

### 4.1 Lexical stream

**Fig 1:**
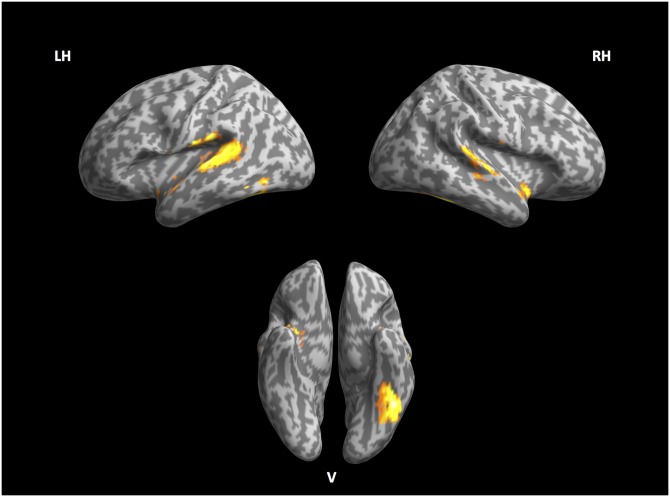
Lexical stream. Left, right and ventral view of inflated cortex plot of the lexical stream.

Table 7 lists the areas that show significant activity with regard to the word-based perplexity regressor. This network is displayed in Fig 1 and it encompasses large portions of the left inferior temporal gyrus (l-ITG) including the fusiform gyrus (l-FG). Both left and right posterior banks of the superior temporal gyrus (rl-STG) are part of this network, together with parts of left anterior superior temporal gyrus.

**Table 7:** Significant effects of lexical stream perplexity.

Region	MNI	size	t-value max
left inferior temporal gyrus—fusiform gyrus	−44 −48 −14	924	5.92
left posterior superior temporal gyrus left middle temporal gyrus	−56 −26 8 −58 −22 2	1876	6.29 4.59
left anterior superior temporal gyrus (TP)	−40 2 −16	121	5.72
right posterior superior temporal gyrus & sulcus	64 −10 −2	1436	4.72

### 4.2 Syntactic stream

**Fig 2:**
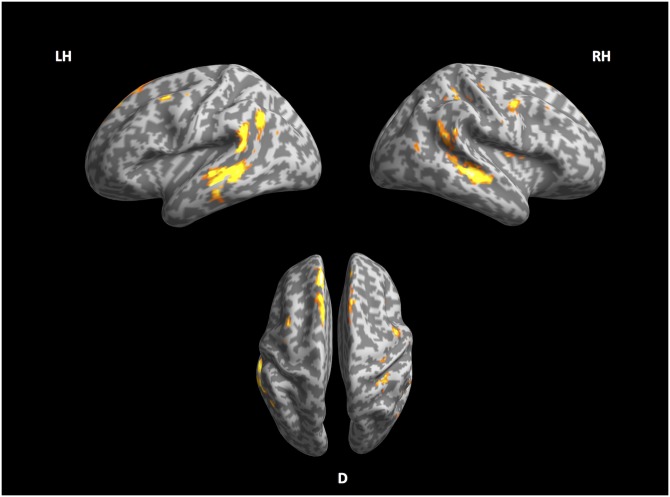
Syntactic stream. Left, right and ventral view of inflated cortex plot of the syntactic stream.

Fig 2 shows the cortical network corresponding to PoS-based perplexity. These include the left middle temporal gyrus and sulcus (l-MTG and l-MTS) and right middle temporal sulcus (r-MTS). The bilateral precentral sulcus is also activated. Large portions of the superior frontal gyrus are also sensitive to these regressor. The list of areas with the coordinates of their activation peaks can be found in Table 8.

**Table 8:** Significant effects of syntactic stream perplexity.

Region	MNI	size	t-value max
left middle superior frontal gyrus	−6 34 56	1549	6.50
left precentral sulcus	−42 6 54	267	6.19
left middle temporal gyrus & sulcus	−64 −50 14	1715	5.64
left and right cerebellum 9	−20 −46 −36	662	5.07
right middle temporal sulcus right angular gyrus right superior temporal sulcus	48 −32 −2 64 −50 24 54 −22 −6	983	5.51 5.49 4.71
right putamen right amygdala	24 −2 6 22 2 −8	649	6.04 4.46
right precentral sulcus	52 −2 46	119	4.31

### 4.3 Phonological stream

**Fig 3:**
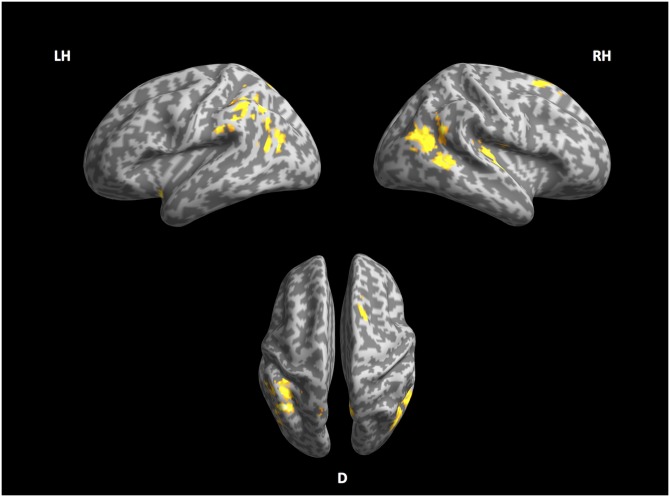
Phonological stream. Left, right and ventral view of inflated cortex plot of the phonological stream.

Fig 3 and Table 9 refer to the network of the phonological stream. This stream involves the right Heschl’s gyrus (r-HG), and right superior frontal gyrus (r-SFG) together with the supplementary motor area (r-SMA). Other areas activated to this contrast are the left insula, the left angular gyrus (l-AG), the left inferior parietal lobule (l-IPL) and bilateral portions of the middle temporal gyrus (rl-MTG). The phonological stream was the only level where perplexity and surprisal did not give comparable results (see supplementary material S1 File). In S1 File we speculate that this may be caused by the way surprisal and perplexity are computed, and we want to point out this unexpected discrepancy here to highlight that the results of the phonological stream should be interpreted with some more caution than the other two streams.

**Table 9:** Significant effects of phonological stream perplexity.

Region	MNI	size	t-value max
left insula	−36 8 −18	123	4.75
left angular gyrus left inferior parietal lobule	−40 −56 40 −42 −44 44	1507	4.78 4.35
left posterior mid temporal gyrus	−42 −64 16	137	3.71
right Heschl’s gyrus right Heschl’s gyrus	50 −12 4 40 −24 12	443	4.63 4.46
right posterior mid temporal gyrus right angular gyrus	42 −64 16 56 −56 24	950	4.14 4.09
right superior frontal gyrus—SMA	20 16 62	202	4.49

### 4.4 Overlap

The results obtained from lexical, syntactic and phonological perplexity allowed us to analyse possible overlap at the cortical level for all three regressor streams. Table 10 contains the name of regions whose activity is significantly explained by more than one regressor. We computed the overlap between the activation maps relative to the lexical and the syntactic stream, the lexical and phonological streams, and the syntactic and phonological stream by taking only those voxels that are significantly activated for both regressors as described in the sections above. This is equivalent to performing a conjunction analysis, more specifically a test of the ‘conjunction null’, effectively looking for statistical significance in both contrast maps as testing a logical AND [39]. We also looked for voxels shared by all three streams.

**Table 10:** Areas of overlap between the streams regressors.

Streams	regions	∼MNI coordinates
Syntax ∩ Lexical	right STS left posterior MTG	54 −25 0 −58 −51 11
Syntax ∩ Phonology	right AG left AG	59 −56 29 −53 −64 31
Lexical ∩ Phonology	right middle STG	44 −29 13
Syntax ∩ Lexical ∩ Phonology	∅	∅

**Fig 4:**
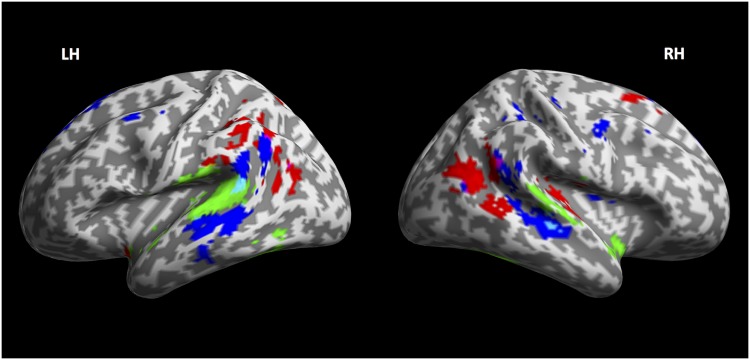
Streams comparison and overlap. Inflated cortex view of the maps of the lexical (green), syntactic (blue) and phonological (red) streams. In this view the overlap between lexical and syntactic streams is particularly evident in the right Middle Temporal Lobe and in the left posterior Superior Temporal Gyrus (cyan). Overlap between syntactic and phonological streams is also evident in the bilateral Angular Gyrus (violet).

It is interesting to note that although some degree of anatomical overlap exists among all possible pairs of regressors, there is no area that is significantly activated for all three streams together. What is also worth noting is that the lexical and syntactic regressors are both processed in the posterior portions of the bilateral middle temporal gyrus, bordering the posterior superior temporal gyrus. Moreover, lexical information and phonology seem to share activity in the central banks of the superior temporal gyrus, but not directly in Heschl’s gyrus, which confirms its selectivity for the phonological stream only. The overlap regions are shown in cyan (lexical and syntactical streams) and in violet (syntactic and phonological streams) in Fig 4.

## 5 Discussion

The results reported above outline a set of cortical networks that are separately activated for each of the three types of information under investigation—lexical, syntactic and phonological–– confirming the hypothesis that language processing can indeed be decomposed into different streams corresponding to different subdivisions of the language network. No area shows selectivity for all three streams, and only limited sets of voxels show overlap between pairs of streams.

### 5.1 Division of labour in the temporal cortex

The temporal lobe shows a distribution between the three streams that sees the lexical information primarily concerning the infero-lateral regions, syntactic information the mid-lateral regions and phonological information finding its hub in the middle superior temporal plane. Areas posterior to the perisylvian cortex, between AG, SMG and IPL, display a similar gradient, with more rostral voxels selecting lexical information and more caudal ones phonological information, with selectivity to PoS-related information in the middle.

### 5.2 Phonological stream

The phonological stream seems to involve activity in the temporal cortex only in regions close to the transverse gyrus (Heschl’s gyrus), especially in the right hemisphere. This area is the central hub of auditory processing [40]. Although the phonological regressor is built on a level of annotation that is close to the actual perceptual structure of the words, it is not directly built on the auditory properties of the stimulus, making these observed results both surprising and interesting. In addition to this, the phonological stream activates the supplementary motor area (SMA), which has been suggested to be involved not only in speech production [41], but also in speech processing [42, 43]. Phoneme perplexity did not return activation in the premotor cortex, an area that has been associated with speech production and perception. Nonetheless, activation of the premotor cortex in response to phonological load is not a general finding in the literature. Tremblay and colleagues [44, 45] have suggested that premotor cortex activation during speech processing may only be observed under tasks presenting particularly difficult conditions. In line with this position, Sato and colleagues [46] have shown that stimulating the premotor cortex only has an effect on a complex speech perception task. Similarly, premotor activity has been found to be modulated by syllable complexity during speech production but not during speech perception [44].

### 5.3 Lexical and syntactic streams

#### 5.3.1 Middle temporal gyrus

Studies by Dronkers and colleagues [47] have suggested that the posterior MTG plays a role in retrieving lexical and syntactic properties of incoming words from long-term memory. Hagoort [3] suggests that MTG might be important for the retrieval of the syntactic frames (as well as other lexical information) from the mental lexicon which are then combined in the left inferior frontal cortex [48]. This intuition is corroborated by our results, which show that activity in this area, although mainly explained by the syntactic regressors employed in our analyses, displays an overlap between the syntactic regressor and the lexical stream. The work of [49] showed that Dutch noun-verb homonyms (grammatical category ambiguity) increased activity in the posterior MTG. This study also reports that grammatically ambiguous sentences activated not only the posterior MTG but also the precentral gyrus, an area that we also observe in our analysis.

Although PoS perplexity intends to model syntactic processing and appears to be a reasonable correlate of the syntactic stream, our analysis shows only a marginal correlation between this regressor and the activity observed in anterior temporal cortex. Studies from Pallier [50], Obleser [51] and Brennan [52] suggest that this region is sensitive to the syntactic complexity of the input sentence. Brennan and colleagues used a similar experimental paradigm to the one adopted in this paper. They had participants listen to a segment of a novel (Lewis Carroll’s *Alice in Wonderland*) and looked for areas of which the activity correlates with the number of so-called syntactic building operations at each time point, representing the number of non-terminal phrases that are completed by the presentation of each word. This measure is based on a hierarchical treatment of syntax, whereas we intended to model syntactic processing in a purely sequential manner. These two results can be reconciled by considering syntactic processing as underpinned by both sequential probabilistic machinery (captured by PoS-perplexity) and hierarchical structure building.

#### 5.3.2 Inferior temporal cortex

Activity in the inferior and lateral portions of the left temporal cortex are better explained by the lexical regressor and are likely to be a central hub of the lexical stream. [25] observed the same result using the same dataset, nonetheless interpreting it as activity in the visual word form area (VWFA) [53]. Their explanation is that word prediction can account for the pre-activation of the upcoming word form in the sequential sentence processing. This explanation is not the only possible one. For instance, Price [54] points out that the cortical region corresponding to VWFA is active in normal subjects also during tasks that do not engage visual word form processing. On the other hand, if activity in left Inferior Temporal cortex and specifically in VWFA truly reflects word form prediction, we would have expected also phonological perplexity to show selectivity in this region. Phonological perplexity, computed on the phonemic structure of each single word, seems intuitively a closer proxy for the form of a word. Although computed on the phonemic transcription of the words, the relation between phoneme and grapheme in Dutch is at least somewhat regular, making visual and phonemic structure intuitively close. Nonetheless, as explained above, this model does not predict activity in ITC better than word-based perplexity. This suggests indeed that the coupling between the later regressor and activity in this region reflects lexico-semantic rather than word form information.

That the lexical regressor, computed in terms of trigram statistics in the co-occurrences of words, is a correlate of lexical semantic processing is strengthened by the outcome of a meta-analysis of 120 functional neuroimaging studies [55, 56]. The meta-analysis showed that lateral and ventral temporal cortex is among the main nodes of the semantic processing network. This interpretation is supported by studies that reported consistent correlation between lexical semantic models and brain activity in ventro-temporal cortex [57, 58].

### 5.4 Left inferior frontal gyrus

None of the perplexity-based regressor returned significant activation in the left inferior frontal gyrus (l-IFG). While l-IFG is an important node in the neural language network, its involvement and potential role during language comprehension has been the subject of considerable debate.

One line of work starting with Thompson-Schill et al. [59] has argued that the role of this area is better characterized as a general, not language specific one, and involved in ‘selection’ or—more generally—‘cognitive control’. Another approach has stressed the role of the area in structural processing, both in a hierarchical and sequential fashion [60, 61]. Nonetheless, not all results seem to support this view. Brennan and colleagues [52, 62], for instance, found that syntactic complexity did not correlate with l-IFG activity, which seems at odds with some previous findings that did observe l-IFG activation in response to syntactically hard to parse sentences. The fact that l-IFG was not detected in Brennan’s work and in the work presented in the present paper might be due to methodological reasons. Both Brennan and we used naturalistic stimuli and correlation between brain imaging data and stimuli properties (stochastic in our case, hierarchically structural in the case of Brennan and colleagues). The literature advocating the role of l-IFG in processing is dominated instead by paradigms comparing carefully constructed sentences, for instance syntactically ambiguous vs. unambiguous [49], or grammatical vs. ungrammatical [63, 64].

In the scope of the present paper, we cannot draw any strong conclusion regarding l-IFG on the basis of its ‘non-activation’.

## 6 Conclusions

In this paper we have shown that the stochastic sequential processing paradigm is indeed a powerful formalism able to predict neurobiological correlates in areas belonging to the language processing network, also when applied to sub-lexical (phonemic) and syntactic (part of speech) levels. Previous work has demonstrated that language processing can be characterized as a stochastic process computed on sequences of words, and that measures of stochastic perplexity are good predictors of brain activity in language sensitive cortical areas.

Word-based (lexical), part of speech-based and phoneme-based perplexity distinctively predict activity in largely separated cortical networks in the temporal, inferior parietal and perisylvian cortex of subjects listening to naturalistic linguistic input.

These results appear to confirm the intuition that language is processed in parallel by distinct networks sensitive to different sources of information, including at least the ones tested here: phonological, lexical and syntactic.

## Supporting information

S1 File Surprisal-based analysis. (PDF)

S2 File 1-gram frequency analysis. (PDF)
