# Correction: Using stochastic language models (SLM) to map lexical, syntactic, and phonological information processing in the brain

**DOI:** 10.1371/journal.pone.0213450

**Published:** 2019-02-28

**Authors:** The PLOS ONE Staff

## Notice of republication

This article was republished on February 21, 2019 to correct for errors in the Data Availability statement introduced during the typesetting process. The publisher apologizes for the errors. Please download this article again to view the correct version.

## References

[1] Lopopolo A, Frank SL, van den Bosch A, Willems RM (2017) Using stochastic language models (SLM) to map lexical, syntactic, and phonological information processing in the brain. PLoS ONE 12(5): e0177794 10.1371/journal.pone.0177794 28542396 PMC5436813

