# Supplementary material for: Using stochastic language models (SLM) to map lexical, syntactic, and phonological information processing in the brain
Source: PLoS One. 2017 May 18;12(5):e0177794. doi: 10.1371/journal.pone.0177794 (PMC5436813; doi:10.1371/journal.pone.0177794)
Supplement: S1 File — (PDF) [file pone.0177794.s001.pdf]

S1. Surprisal-based analysis

In this appendix we present the results of analyses that employed surprisal instead of perplexity as stochastic measures to compute the stream-wise regressors. The data and procedure are the same as described in the body of the paper. S! Table 1 contains the cluster size thresholds following the Slotnick's procedure. S1 Tables 2 and 3 report the results for the lexical and syntactic streams. To note is the fact that these results do not differ substantially from those observed to perplexity (reported in Section 4 of the main paper). However, when surprisal is computed on the phonemic structure of words, it does not return any significant cluster of activation across the brain after application of the cluster size threshold.

**Region**	**Cluster size**
Word-based surprisal	92
PoS-based surprisal	97
Phoneme-based surprisal	86

S1 Table 1: Cluster size thresholds for the surprisal-based regressors

**Region**	**MNI**	**size**	**t-value max**
left inferior temporal gyrus - fusiform gyrus	−46 −46 −18	720	6.57
left posterior superior temporal gyrus	−58 −26 12	944	5.20
left anterior superior temporal gyrus (TP)	−40 2 −16	394	4.97
left amygdala	−28 −6 −10		3.95
right posterior superior temporal gyrus	66 −28 12	156	4.39
right amygdala	30 −2 −14	872	4.23
right anterior superior temporal gyrus (TP)	48 10 −16		4.03

S1 Table 2: Significant effects of lexical stream surprisal

**Region**	**MNI**	**size**	**t-value max**
left middel temporal gyrus - middle temporal sulcus	−58 −38 −2	2012	6.73
left angular gyrus	−58 −52 30		6.06
left precentral sulcus	−44 4 54	313	6.85
left middle superior frontal gyrus	−6 38 52	2017	6.91
left and right cerebellum 9	−12 −52 −38	689	5.03
left inferior parietal cortex	−30 −22 54	284	3.37
right middle temporal sulcus	50 −32 −2	1642	5.56
right putamen	26 −2 8	627	5.72
right precentral sulcus	52 −2 46	360	4.93

S1 Table 3: Significant effects of syntactic stream surprisal

The large overlap between the two information measures is to be expected given that perplexity and surprisal capture the same type of information, namely the deviation between the expected and the observed next item (word, grammatical category or phoneme) given the previous context. Perplexity is the exponential transformation of surprisal, therefore it magnifies the higher probabilities and minimizes the lower values. This might be the most likely reason for the discrepancies between the results regarding the phoneme-based analysis. Considering (a) that the per-word regressor is computed as the average over all phonemic transitions per word, (b) that surprising phonemic transitions are relatively rare as compared to the majority of phonotactically predictable transitions, and (c) that perplexity is an exponential amplification of surprisal, surprising transitions are retained better in the per-word averages with perplexity than with surprisal.
