# Supplementary material for: Using stochastic language models (SLM) to map lexical, syntactic, and phonological information processing in the brain
Source: PLoS One. 2017 May 18;12(5):e0177794. doi: 10.1371/journal.pone.0177794 (PMC5436813; doi:10.1371/journal.pone.0177794)
Supplement: S2 File — (PDF) [file pone.0177794.s002.pdf]

S2. 1-gram frequency analysis

The analyses described in the main paper used as regressors of interest perplexity obtained from 3-gram language models computed on words, PoS, and phonemes. Conversely, the effect of word, PoS and phoneme 1-gram frequencies were factored out by using them as regressors of no interest. In this paragraph we report the results of additional analyses on the effect of these 1-gram. Analysis consisted in using these 3 measures as regressors of interest in turn following the fMRI analysis pipeline also adopted for the perplexity regressors. Of the 3 frequencies, only lexical frequency returns significant clusters of activation, consisting in a large area of activity centred in the bilateral middle cingulate cortex and the right precuneus. Activity relative to lexical frequency also touches areas such as the left precentral sulcus, parts of the bilateral inferior parietal touching into bilateral posterior superior temporal gyrus, an area, this one, central in the lexical stream as defined by lexical 3-gram perplexity. Overall, the 1-gram and 3-gram measures explain brain activity in different networks, strengthening the intuition that the streams capture processing in line with the predictive brain framework.

**Region**	**MNI**	**size**	**t-value max**
right precuneus	26 −60 30	16982	7.23
right/left mid cingulate cortex	22 −38 36		6.71

S2 Table 1: Significant effects of lexical frequency.
